# Clinical Outcomes and Prognostic Analysis of 101 Patients of Central Neurocytoma: A 10-Year Treatment Experience at a Single Institution

**DOI:** 10.3389/fonc.2022.881460

**Published:** 2022-05-27

**Authors:** Qiongxuan Xie, Bin Xie, Ludi Ou, Min Wang, Ziqing Tang, Yuxiang He, Xiaoyu Yang, Jidong Hong, Zhiping Lyu, Rui Wei

**Affiliations:** ^1^ Department of Oncology, Xiangya Hospital, Central South University, Changsha, China; ^2^ National Clinical Research Center for Geriatric Disorders, Xiangya Hospital, Central South University, Changsha, China; ^3^ Department of Pathology, Xiangya Hospital, Central South University, Changsha, China

**Keywords:** central neurocytoma, atypical neurocytoma, survival outcomes, prognostic factors, treatment, radiotherapy

## Abstract

**Objective:**

Central neurocytoma (CN) is a rare type of tumor that currently lacks an optimal treatment protocol. This study aimed to explore the clinical outcomes of CN in a cohort of 101 patients and identify prognostic factors associated with multiple treatment modalities.

**Methods:**

This monocentric study retrospectively analyzed the clinical data of 101 CN patients who underwent surgical resection. The patients were followed up, and their overall survival (OS) and progression-free survival (PFS) were calculated.

**Results:**

For the entire cohort, the 5- and 10-year OS rates were 88.7% and 82.8%, respectively, and the 5- and 10-year PFS rates were 86.5% and 64.9%, respectively. Of the 82 (81.19%) patients with CN who underwent gross total resection (GTR), 28 (28/82, 34.1%) also received radiotherapy (RT). Of the 19 (18.81%) patients with CN who underwent subtotal resection (STR), 11 (11/19, 57.9%) also received RT or stereotactic radiosurgery (SRS). Compared to STR, GTR significantly improved the 5-year OS (92.4% vs. 72.4%, P=0.011) and PFS (92.4% vs. 60.4%, P=0.009) rates. Radiotherapy did not affect OS in the GTR group (p=0.602), but it had a statistically significant effect on OS in the STR group (P<0.001). However, the OS (P=0.842) and PFS (P=0.915) in the STR plus radiotherapy group were comparable to those in the GTR alone group. Compared to STR alone, STR plus radiotherapy improved the 5-year PFS rate from 25% to 75% in patients with atypical CN (P=0.004). Cox regression models and a competing risk model showed that the removal degree and radiotherapy were independent prognostic factors for survival. With improvements in modern radiotherapy techniques, severe radiotherapy toxicity was not observed.

**Conclusion:**

Our findings support the use of GTR whenever possible. Radiotherapy can improve the prognosis of patients who undergo STR, especially in atypical CNs having a higher tendency to relapse. Close imaging follow-up is necessary. Our findings will help clinicians to select optimal, individualized treatment strategies to improve OS and PFS for patients with CN.

## Introduction

Central neurocytoma (CN) is a rare central nervous system tumor that occurs in adolescents and accounts for approximately 0.1 to 0.5% of intracranial tumors (1–4). Spinal cord dissemination is rare (5). In 1982, Hassoun identified the first two cases of CN, coining the term to describe intra-lateral ventricular tumors with typical features of neuronal differentiation (6). According to the 2006-2014 epidemiological survey of the Central Brain Tumor Registry of the United States (CBTRUS), the annual incidence of CN is 0.022 (0.021–0.024), and is slightly lower in men than in women (7). The majority of CNs (approximately 70%) occur in young people aged 20 to 40 years and are found in the third and lateral ventricles (3, 8). Patients initially clinically present with headache and visual disturbance caused by intracranial hypertension and obstructive hydrocephalus (9). CNs originate from neural stem cells with bidirectional differentiation potential around the ventricles. They are neuronal and mixed neuronal glial tumors which are classified as grade 2 in accordance with the 2021 World Health Organization classification (10). Although most CNs are well differentiated and generally have a good prognosis, some exhibit malignant biological behavior (11, 12).

Where possible, complete surgical resection is the preferred treatment modality for CN; otherwise, incomplete surgical resection followed by adjuvant radiotherapy (RT) or stereotactic RT to control any residual tumor and reduce tumor recurrence is considered (2, 9). With clinical, radiological, and pathological developments in recent years, our understanding of CN has improved; however, due to the rarity of CN, the current selection of treatment options is informed only by case reports, small-sample retrospective analyses, and meta-analyses, and there are no prospective studies with large samples and prolonged follow-up (2, 13, 14). Furthermore, the treatment options for recurrent tumors are still controversial (15, 16). Consequently, there is still much debate about surrounding the best way to treat this disease.

In this study, we collected clinical information from 101 patients with CN from 2010 until 2020 including basic characteristics, treatment patterns, and long-term survival outcomes. We then analyzed this information and reviewed the literature in order to gain insight into the characteristics of CN and identify optimal treatment modalities and prognostic factors.

## Methods

### Patient Data

Our study cohort included 101 eligible patients admitted to Xiangya Hospital, Central South University from April 2010 to September 2020 with pathologically confirmed CN. None of the patients had received any previous RT. The records of all eligible patients were sufficiently comprehensive to be analyzed. Patients with an unclear pathological diagnosis, a second primary tumor, or any other malignancies were excluded. Eligible patients were followed up as outpatients or by telephone. Typically, clinical and neuroimaging evaluations were performed at 3 months after discharge and every 6 months after stabilization. Postoperative magnetic resonance imaging (MRI) was performed every 6 months for the first 2 years and annually thereafter. The median follow-up time for our cohort was 47.90 (range 0.03-132.23) months. Patients’ medical records, including their demographic information, treatment modality, and survival outcomes, were collected. This retrospective cohort study was approved by the ethics committee of Xiangya hospital. As this was a retrospective study, patient consent was not required.

### Baseline Data and Variables

Baseline data included patient demographic characteristics, pre-RT treatment, tumor characteristics, and RT parameters. Pre-RT treatment information included the extent of tumor resection. Tumor characteristics included the tumor location and volume.

### Treatment Modalities

All patients underwent surgery. Based on a review of the patients’ surgical records and postoperative imaging, the first surgical resections were divided according to scope into gross total resection (GTR) and subtotal resection (STR). GTR was defined as 100% gross resection of the tumor or no indication of residual tumor in early postoperative imaging data, and STR was defined as partial or incomplete resection or some evidence of residual tumor in early postoperative imaging data. The primary surgical methods employed were the transcortical approach and the interhemispheric transcallosal approach. In children, the surgical approach is occasionally used to cut the dura into the lateral ventricle.

Radiotherapy uses techniques included intensity-modulated radiation therapy (IMRT), helical tomotherapy (HT), and Gamma Knife. IMRT and HT are both forms of conventional fractionated external-beam radiation therapy (EBRT), and Gamma Knife is a type of stereotactic radiosurgery (SRS). During EBRT, patients were positioned and secured with a thermoplastic head and shoulder mask. Then, the CT and MRI images are imported into Eclipse TPS (Varian Medical Systems Inc., Version 11.0.31) and combined to enable the clinician to delineate the target area. The gross tumor volume (GTV) and the organs at risk (OAR) were outlined by an experienced radiologist and confirmed after discussion with radiation oncologists. The clinical target volume (CTV) was the defined as grossly tumor volume plus subclinical microscopic tumors, and the planning target volume (PTV) was defined as the CTV plus set-up error. The GTV was expanded 1-2 cm to form the CTV and the CTV was expanded 0.3 cm to form the PTV. The radiotherapy treatment was delivered 5 days a week, once a day from Monday to Friday. The median radiation dose was 54.54Gy (range: 46–61.60Gy) and the single dose ranged from 1.8–2.14Gy.

The tumor volume was calculated as (length * width * height/2) or (length * width^2^/2) (17, 18). The restriction of the organ at risk dose was determined according to the ESTRO-ACROP consensus guideline (19). The HT protocol uses tomotherapy Hi-Art Software (Version 2.0.7) (Accuray, Madison, WI, USA), and the IMRT protocols use Varian Eclipse TPS (Varian Medical System, USA). For patients treated with Gamma knife, they were treated with the Leksell-B Gamma Knife. Dose planning is performed using the Gamma Knife Treatment Planning System.

### Statistical Analysis

Overall survival (OS) was defined as the time interval from the date of pathological diagnosis to the date of death or the last follow-up assessment. Progression-free survival (PFS) was defined as the time interval from the date of pathological diagnosis to the date of tumor recurrence or the last follow-up assessment. Local recurrence was defined as progressive enlargement of the target lesions. The primary endpoints were OS and PFS. The Kaplan–Meier with log-rank test method was adopted for survival analysis. Categorical variables were analyzed using univariate and multivariate logistic analysis.

Three multivariable-adjusted Cox proportional-hazards regression models were used to calculate hazard ratios (HRs) with 95% confidence intervals (CIs). Model 1 was adjusted for sex, type, size, age, and karnofsky performance status (KPS) score before surgery. Model 2 was further adjusted for extent of resection to estimate the effect of surgery on survival outcome; however, no adjustment was made for postoperative radiotherapy (due to the potential mediation of radiotherapy). Model 3 was further adjusted for postoperative radiotherapy. Postoperative complications are a fundamental prognostic factor for patient death. If a patient died due to early postoperative complications, then they could not undergo further RT and long-term survival could not be observed; therefore, a Fine and Gray’s competing risk regression model was used to further assess the impact of RT on survival. Competing factors included death from acute postoperative complications and death from oncological factors. In this study, IBM SPSS software, version 26.0 (IBM Corporation, Armonk, NY, USA) was used for statistical analyses, and R version 4.1.1. was used for mapping and statistical analyses. Statistical significance was defined as a P-value of < 0.05.

## Results

### Patient Characteristics

Of the 101 patients with CN, 58 were male and 43 were female. The age of onset ranged from 10 to 59 years, and the mean age was 31 years. The main clinical manifestations and symptoms were headache (n=72), loss of vision (n=18), and nausea and vomiting (n=17). The median interval from symptom onset to diagnosis was 5.9 months. The patients’ baseline and tumor characteristics are presented in **Table 1**.

**Table 1:** Characteristics for patients with CNs.

Characteristic	Value
**Sex**	
Male	58 (57.4%)
Female	43 (42.6%)
**Age**	
Range	10-59
Mean ± SD	31.69 ± 11.591
<30 y	55 (54.5%)
≥30y	46 (45.5%)
**Type**	
Typical	36 (35.6%)
Atypical	65 (64.4%)
**KPS before surgery**	
≥70	94 (93.1%)
<70	7 (6.9%)
**Location**	
The left ventricle	42 (41.6%)
The right ventricle	25 (24.8%)
Third ventricle	6 (5.9%)
Two or more ventricles	28 (27.7%)
**Size of tumor**	
Maximum diameter of tumor mean ± SD (range)	5.11 ± 1.62 (0.5-9.6)cm
Maximum diameter of tumor>5cm	52 (51.5%)
Maximum diameter of tumor ≤ 5cm	49 (48.5%)
The tumor volume mean ± SD (range)	49.86 ± 38.541 (0.005-220.5)cm^3^
**Initial Symptoms**	
Dizziness and headache	72 (71.3%)
Visual deficit	18 (17.8%)
Nausea and vomiting	17 (16.8%)
Memory disturbance	8 (7.9%)
Disorders of consciousness	8 (7.9%)
Limb weakness	7 (6.9%)
Unsteady walking	6 (5.9%)
Limb twitching	4 (3.9%)
Tinnitus	3 (2.9%)
Slow response	2 (1.9%)
Limb weakness	2 (2.0%)
Hearing loss	1 (0.9%)
Seizures	1 (0.9%)
Confusion	1 (0.9%)
Syncope	1 (0.9%)
Blindness	1 (1.0%)
Catarrhal symptoms	1 (0.9%)
Numbness	1 (0.9%)
Diplopia	1 0.9%)
Insomnia	1 (0.9%)

**Figure 1:**
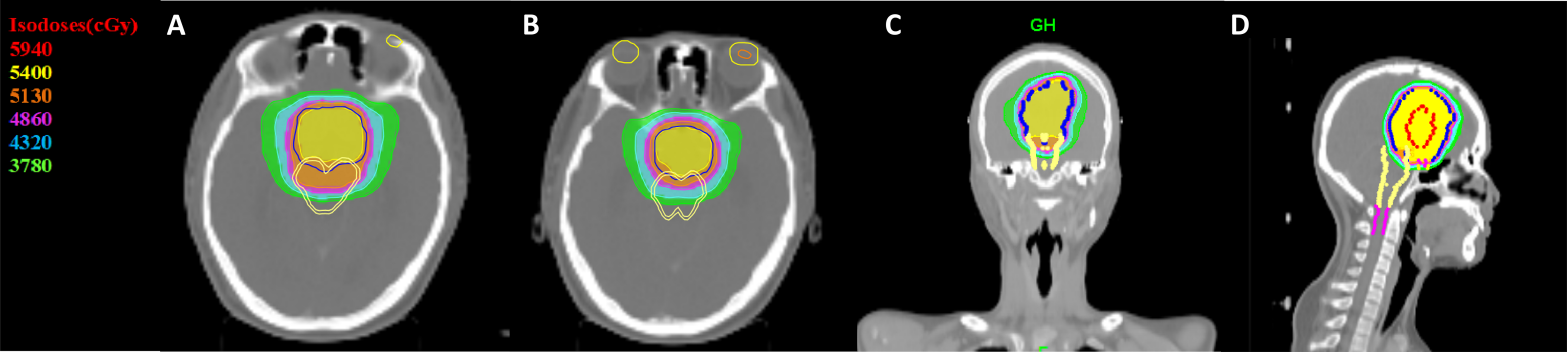
Dose distribution in a CN patient treated with IMRT. Color-wash areas: 59.40Gy (red), 54.00Gy (yellow), 51.30Gy (orange), 48.60Gy (purple), 43.20Gy (blue), 37.80Gy (green). CN, central neurocytoma; HT, Helical tomotherapy. **(A, B)** refer to cross section CT, **(C)** refer to coronal CT and **(D)** refer to sagittal CT.

Of the 101 patients in this study, 13 died (12.87%) and 88 (87.12%) survived. No patients had distant metastases. The different treatments modality are presented in **Table 2**. A total of 38 patients received adjuvant radiotherapy and 1 patient underwent Gamma Knife SRS. Of the 38 patients treated with EBRT, 32 received IMRT, 4 received conventional 3D conformal radiotherapy (3DCRT) and 2 received HT. The IMRT plan has been proven by many studies to have good conformity and homogeneity. Compared with IMRT, the dose gradient of HT is steeper and the dose gradient is better. In our study, only 2 patients received HT treatment after surgery. **Figure 1** shows the dose distributions of patients who were treated with HT. In all instances, the exposure of the brainstem was within the safe dose.

**Table 2:** The treatment modality for patients with CNs.

Treatments	Total	%
**Surgical approach**		
Transcortical	56	55.4
Interhemispheric transcallosal	44	43.6
Transdural entry into the ventricles	1	0.9
**GTR**	82	81.2
GTR alone	54	53.5
GTR+RT	28	27.7
**STR**	19	18.8
STR alone	8	7.9
STR+RT	10	9.9
STR+SRS	1	1.0

GTR, gross total resection; STR, subtotal resection; RT, radiotherapy; SRS, stereotactic radiotherapy.

### Survival According to Patient Baseline Characteristics

Patients with KPS≥70 had better OS than patients with KPS<70 (P=0.001). For the KPS ≥70 group, the 5-year OS and PFS rates were 91.2% and 88.9%, respectively, and for the KPS < 70 group the corresponding values were 51.4% and 51.4%, respectively (P=0.001). Female patients had worse OS than male patients (P=0.038). No statistically significant difference was observed in OS between patients older than 30 years of age and patients aged 30 years or younger (P=0.589). Patients with a tumor diameter ≤ 50 mm had better OS than those whose tumor diameter was > 50 mm (P=0.048).

### Survival According to Treatment Method

For the cohort as a whole, the 5- and 10-year OS rates were 88.7% and 82.8%, respectively, and the 5- and 10-year PFS rates were 86.5% and 64.9%, respectively. A total of 82 patients underwent GTR, and the other 19 patients had STR. For the patients who underwent GTR, the 5-year OS rate was 92.4% and the median survival time was 124.43 months. For the patients who underwent STR, the 5-year OS rate was 72.4% and the median survival time was 83.03 months (P=0.011). The 5-year PFS rate for GTR with or without RT, the STR with or without RT were 92.4% and 60.4% (P=0.009). The mean PFS duration for the GTR group was significantly longer than that for the STR group (117.61 ± 4.10 months vs. 83.75 ± 15.61 months, P=0.009). The 10-year OS rates for GTR+RT and GTR alone were 96.0% and 90.5%, respectively, and RT was shown not to have a significant impact on 10-year OS (P=0.602). The 10-year OS rates for STR+RT and STR alone were 66.7% and 29.2%, respectively, and RT was shown to have a significant impact on 10-year OS (P<0.001). The 10-year OS rate in the GTR+RT group was not significantly better than that in the STR+RT group (96.0% vs. 66.7%, P=0.704). The 10-year PFS rates in the GTR+RT and STR+RT groups were 96.0% and 83.3%, respectively, with no statistically significant difference observed (P=0.741). The 10-year OS rates for GTR without RT and STR+RT were 90.5% and 66.7%, respectively, with no statistically significant difference observed (P=0.842). The 10-year PFS rates for GTR without RT and STR+RT were 90.5% and 83.3%, respectively, with no statistically significant difference observed (P=0.915).

### Survival According to Pathological Type

**Figure 2:**
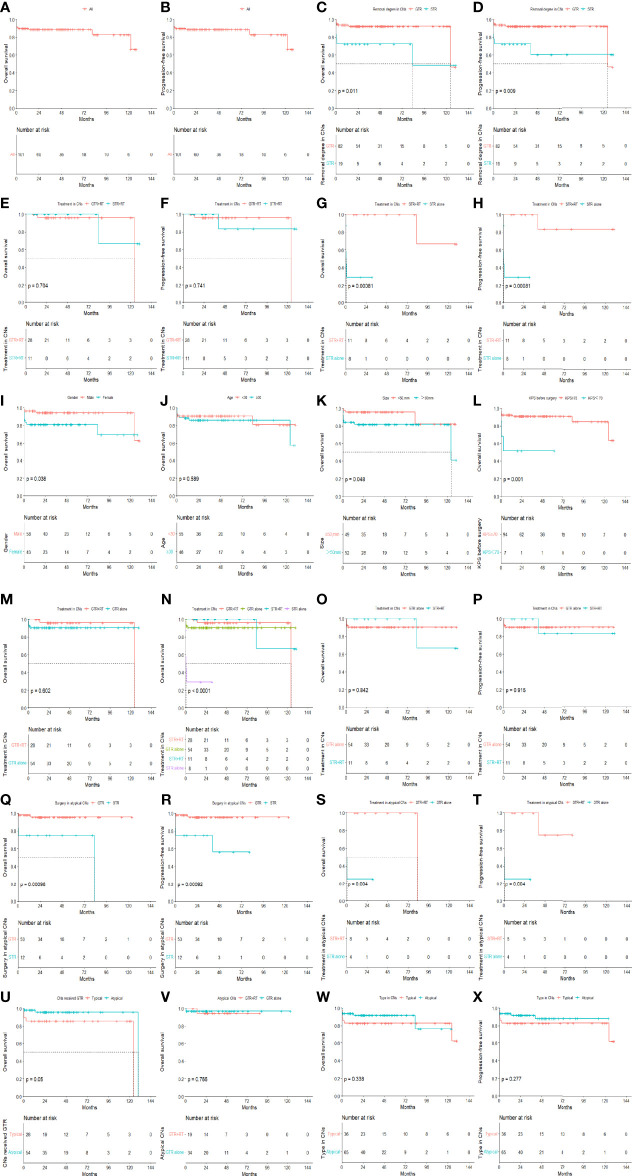
The OS and PFS in all patients **(A, B)**. The effects of removal degree on OS and PFS in all patients **(C, D)**. The effects of removal degree on OS and PFS in all patients treated by RT **(E, F)**. The effects of radiotherapy on OS and PFS in patients treated by STR **(G, H)**. OS curves for patients with different baseline conditions in terms of gender, age, tumor size and KPS **(I–L)**. The effects of RT on OS in patients treated by GTR **(M)**. OS curves of CN patients with different treatments **(N)**. OS and PFS in the GTR alone group compared to the STR with radiotherapy group in all CNs **(O, P)**. The effects of removal degree on OS and PFS in atypical CNs **(Q, R)**. The effects of RT on OS and PFS in atypical patients treated by STR **(S, T)**. OS curves for typical versus atypical patients in GTR **(U)** groups. The effects of RT on OS in atypical patients treated by GTR **(V)**. OS **(W)** and PFS **(X)** curves of different type in all patients.

There was no statistically significant difference in OS (P=0.338) or PFS (P=0.277) between patients with typical CN and atypical CN. In the atypical CN group, there was a significant difference in the mean OS and PFS durations between patients who underwent GTR and those who underwent STR (mean OS duration ± SE: 119.04 ± 62.34 months vs. 62.34 ± 11.95 months, P<0.001, mean PFS duration ± SE: 119.04 ± 62.34 months vs. 53.092 ± 10.94 months, P<0.001). However, there was no statistically significant difference in OS between patients with atypical CN who received GTR+RT and those who received GTR alone (P=0.765). For patients with atypical CN who underwent STR, there was a statistically significant difference in OS between those who received adjuvant RT and those who did not (83.03 ± 0.01 vs. 7.95 ± 6.65, P=0.004), and the mean PFS duration for patients who received adjuvant RT was significantly longer than that of patients who did not (70.70 ± 8.66 vs. 7.95 ± 6.54, P=0.004). The results of the univariate analyses are shown in **Figure 2**.

### Prognostic Analysis

**Figure 3:**
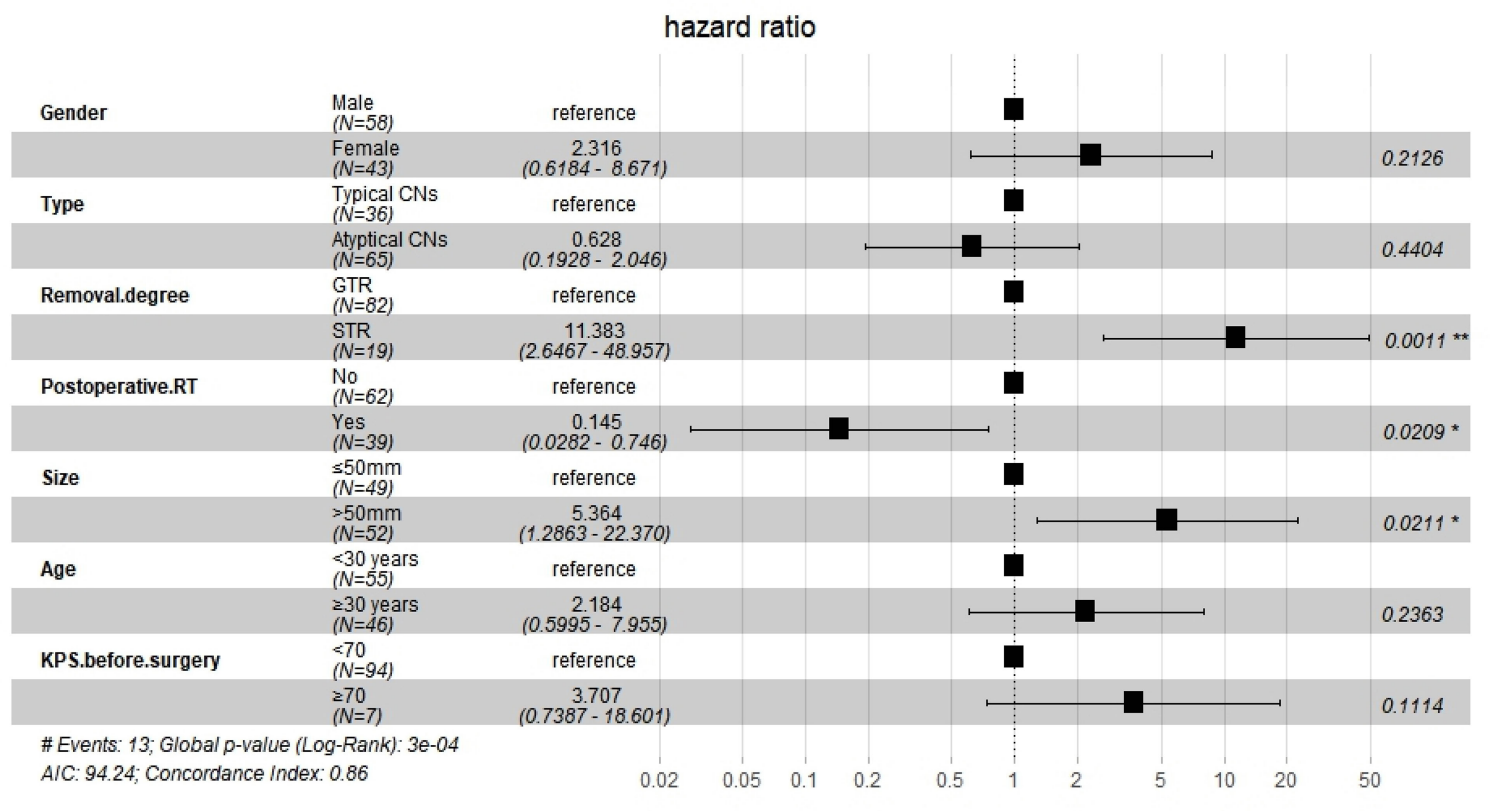
Multivariate Cox regression analysis of factors affecting survival benefit of patients.

After Cox regression analysis, model 1 showed significantly worse OS for women than for men (HR=4.034, 95% CI 1.023–15.917, P=0.046) and for patients with tumors larger than 50 mm (HR=4.323, 95% CI 1.114–16.773, P=0.034). Model 2 showed significantly worse OS for patients who underwent STR than for those who underwent GTR (HR=6.567, 95% CI 1.602–26.920, P=0.009), for patients with tumors larger than 50 mm (HR=5.327, 95% CI 1.256–22.599, P=0.023), and for patients with KPS < 70 before surgery (HR=5.669, 95% CI 1.161–27.679, P=0.032). Model 3 showed that the extent of resection was an independent prognostic factor (HR=11.383, 95% CI 2.674–48.957, P=0.001), and that postoperative RT significantly improved OS (HR=0.145, 95% CI 0.028–0.746, P=0.021). It also showed that tumors larger than 50 mm were associated with significantly worse OS than those measuring less than 50 mm (HR=5.364, 95% CI 1.286–22.370, P=0.021). However, the following were not found to be independent influencing factors associated with a poor prognosis: female vs. male, atypical CN vs. typical CN, age over or equal to 30 years vs. age less than 30 years, and KPS value before surgery. The findings from Model 3 are presented in **Figure 3**.

In the competing risk model, the OS of patients who underwent STR was significantly worse than of patients who underwent GTR (HR=14.514, 95% CI=3.167–66.516, P<0.001), and postoperative RT was associated with significantly improved survival (HR=0.079, 95% CI=0.010–0.602, P=0.014). The findings of Model 3 and the competing risk model are presented in **Table 3**.

**Table 3:** Analysis of two models of different treatment modalities.

Variable	Multivariable Cox hazards model			Competing risk model		
	P value	HR	95%CI	P value	HR	95%CI
**Gender**	0.213	2.316	0.618-8.671	0.350	1.915	0.485-7.566
**Type**	0.440	0.628	0.193-2.046	0.160	0.454	0.075-7.26
**Removal degree**	0.001	11.383	2.647-48.957	0.00057	14.514	3.167-66.516
**Postoperative RT**	0.021	0.145	0.028-0.746	0.014	0.079	0.010-0.602
**Size**	0.021	5.364	1.286-22.370	0.056	5.780	0.959-34.856
**Age**	0.236	2.184	0.600-7.955	0.200	2.377	0.632-8.942
**KPS before surgery**	0.111	3.707	0.739-18.601	0.120	4.030	0.696-23.320

### Failure Patterns and Treatments

In total, 13 patients died. 1 patient who relapsed was treated with Gamma Knife after initial surgery and died after salvage treatment. Surgery and Gamma Knife were chosen as the salvage treatment. Postoperative pathological biopsy revealed a WHO grade IV embryonic tumor. 3 patients died possibly due to tumor-related factors but without imaging, surgery or biopsy performed to prove recurrence: 1 patient died due to acute impaired consciousness and failed to be resuscitated, 1 patient died due to sudden onset of dizziness and a fall, and 1 patient, stabilized in a hemiplegic state, whose symptoms later worsened. The other 9 patients died from postoperative complications: 5 patients eventually died after surgery due to unconsciousness, 1 patient died due to postoperative intracranial hypertension and brain herniation and 3 patients died due to severe post-operative intracranial infections.

### Toxicity

Surgery induced toxicities include infection, hydrocephalus, cerebral hematoma, disturbance of consciousness, language impairment, limb movement impairment, memory impairment, and epilepsy. No serious radiotherapy toxicity. The most common RT toxicity was memory impairment, grade 1-2. Univariate and multivariate logistic analysis showed that, there was no significant association between the surgical approaches, the removal degree and toxicities, respectively. However, postoperative radiotherapy would increase the risk of memory impairment (OR=3.871, 95% CI 1.028-14.577, P=0.045), with a statistically significant difference.

## Discussion

In our study of 101 patients with CN, survival analysis showed that the 5- and 10-year OS rates were 88.7% and 82.8%, respectively, and the 5- and 10-year PFS rates were 86.5% and 64.9%, respectively. This 5-year PFS rate is excellent, and the 5-year OS rate is similar to results reported in previous studies (20–22). The largest meta-analysis to date was performed by Rades et al.; it included 438 patients and found 5- and 10-year OS rates of 91%, a 5-year PFS rate of 73%, and a 10-year PFS rate of 66% (22). Han’s study included 67 patients with CN, which represented the largest single-institution cohort before ours (20). In that study, the 10-year OS and PFS rates were 84.8 ± 6.1% and 79.8 ± 9.5%, respectively, with the latter value significantly different to that from our study. However, in Hallock et al.’s study (23), the 10-year OS rate was 82% and the 10-year local control (LC) rate was 61%, which are comparable to our values. Some of the major reports on survival rates for CN are summarized in **Table 4**. Larger sample sizes and long-term follow-up are needed in the future.

**Table 4:** Survival outcomes of some large retrospective studies on central neurocytomas.

Author/Years	Patients	Treatments	Median follow-up	Survival outcomes	Progression
Samhouri et al. (21)/2021	33 (Multicenter)	GTR+RT:2 STR+RT:17 GTR:7 STR:7	56 mo	5y OS:90% 5y PFS:76%	7
Han et al. (20)/2020	67	GTR+RT:24 STR+RT:9 GTR:31 STR:3	43.5 mo (0.5-135 mo)	10y OS: 84.8± 6.1% 10y PFS: 79.8± 9.5%,	2
Byun et al. (14)/2018	40	GTR+RT:16 STR+RT:10 GTR:9 STR:5	15y	5y OS:97.1% 5y PFS:81.2%	8 (2 typical CNs and 6 atypical CNs)
Chen et al. (24)/2014	63	GTR+RT:24 STR + RT:28 PR + RT:9 Bx + RT:2	69 mo (15-129 mo)	5y OS:96.6% 5y PFS:96.5%	GTR+RT (1/24) STR+RT (1/28) PR+RT (1/9)
Kim et al. (25)/2013	58	GTR+RT:2 STR+RT/RS:11 NTR+RT:1 GTR:22 STR:6 NTR:6 RS:10	119 mo (18-304 mo)	5y OS:91% 10y OS:88%	GTR (2/2) STR+RT (1/11) NTR+RT (1/1) STR (1/6) NTR (1/6)
Vasiljevic et al. (17)/2012	71 (Multicenter)	GTR+RT:2 STR+RT:4 STR+chemo:2 STR+S:6 GTR:41 STR:16	48 mo (6-204 mo)	N/A	GTR (4/43) STR (9/28)
Hallock et al. (23)/2011	19	STR+RT:1 GTR:10 STR:8	104.5 mo (0.75-261.7 mo)	10 y OS:82% 10 y LC: 61%	GTR (1/10) STR (4/8)
Leenstra et al. (2)/2007	45	GTR+RT:6 STR+RT:8 GTR:15 STR:14 Bx+RT:2	10y (1.6–23.4y)	5y and 10y OS:83% 5y LC:67% 10y LC:60%	LR (15/45) GTR (5/15)
Rades et al. (22)/2006	438 (meta-analysis)	CTR+RT:43 ITR+RT:134 CTR:152 ITR:109	44 mo (12–456 mo)	All:5y and 10y OS:91% 5y PFS:73%, 10y PFS:66% CTR+RT: 10y OS:97% 10y LC:87% ITR+RT: 10y OS:89% 10y LC:76% CTR: 10y OS:99% 10y LC:74% ITR: 10y OS:82% 10y LC:35%	N/A
Schild et al. (4)/1997	32	STR+RT:8 GTR+RT:5 GTR:5 STR:14	4.7y (2.3-15.3y)	5y OS:81% 5y LC:79%	N/A

NTR, near-total resection; N/A, not applicable; Bx, biopsy; CTR, complete resection; ITR, incomplete resection.

Surgical resection is generally accepted as the first-choice treatment for CN. Not only can it remove the tumor and reduce the symptoms of intracranial hypertension caused by hydrocephalus, but it can also clarify the nature of the tumor pathology (26). For patients with CN, surgical resection can achieve a 5-year LC rate exceeding 70% (4, 27). In our study, all patients underwent surgery as the first treatment, and there was a statistically significant survival benefit in the GTR group as compared to the STR group in terms of 5-year OS and PFS. Many other studies have suggested that GTR plays a crucial role in the treatment of CN and is associated with a lower CN recurrence rate (17, 22). The feasibility of GTR is influenced by the location, depth, and size of the tumor, and it is not always a practical treatment strategy. However, complete surgical resection should always be performed when possible.

Transcortical or interhemispheric transcallosal approaches are usually used for CN (28, 29). Han et al. showed that there is no significant difference between these surgical approaches in terms of the effect on the patient’s memory (20). The transcortical approach avoids the parasagittal sinus vein, but disrupts the projection fibers of the frontal lobe. The transcallosal approach, with its high flexibility of exposure, is popular due to its smaller incision, but it also entails a higher incidence of disconnection syndrome (28). For large tumors, a transcortical approach is usually chosen; however, this carries the risk of seizures, hemiparesis, aphasia, and memory impairment. For small or medium-sized tumors, an interhemispheric transcallosal approach is usually chosen, which risks the occurrence of bridging vein injury or disconnection syndrome (30, 31). Many investigators prefer the transcallosal approach because of the smaller incision (20). Therefore, the tumor location and characteristics, minimization of blood loss, maximization of the extent of tumor removal, and minimization of complications should all be taken into account when determining the surgical approach to take.

Postoperative RT plays an important role in LC. Since complete resection is not always feasible and CNs tend to occur in young adults, adjuvant RT is now widely accepted as a treatment (2, 11). A meta-analysis by Rades et al. including 438 patients showed that ITR had significantly lower LC rates than CTR alone or in combination with other treatments (22). 5 and 10-year local control rates for ITR alone were 46% and 35%, respectively; however, ITR plus adjuvant RT increased the 5-year local control rate to 83% and the 10-year local control rate to 76%, which are comparable to the rates for CTR with or without RT (22). Several authors (27) have reported that postoperative RT has no effect on OS or PFS in patients who have undergone GTR, whereas it can significantly improve survival for patients who have undergone STR. In Han et al.’s study, there was no significant difference in OS between patients treated with GTR+RT and those treated with STR+RT (P=0.263) (20). A study from Mayo Clinic showed postoperative RT improved LC in patients with atypical CN with a high risk of recurrence (2). Our study produced similar results. In our study, there was no significant OS benefit in the GTR+RT group over the GTR alone group (P=0.602), but there was a significant OS benefit in the STR+RT group over the STR alone group (P<0.001). However, the OS (P=0.842) and PFS (P=0.915) of the STR+RT and the GTR without RT groups were comparable.

Regarding the effect of different RT technologies in the treatment of CNs, an international multicentric study did not observe any differences in survival for CNs associated with RT devices (21). Paek et al. suggest that more sophisticated radiation techniques should be used to reduce the subsequent sequelae in normal brain tissue (32). Modern RT techniques such as IMRT, volumetric arc radiation therapy (VMAT), and HT protect the surrounding normal tissue well, but HT increases low dose volume. The impact on the late toxicities in young people treated by HT needs to be evaluated in the long term. In our study, no grade 3 or 4 radiotoxicity was observed in patients who had been treated using modern RT techniques.

Adjuvant RT can lead to side effects such as memory impairment and secondary tumors (33, 34). Chen et al. reported that a common toxicity is temporary memory impairment (34). In our study, multivariate analysis showed that RT increased the risk of memory impairment (OR=3.871, 95% CI 1.028-14.577, P=0.045). However, many studies have shown that surgery is associated with a risk of memory impairment in patients with lateral ventricular tumors (35, 36). Schild et al. reported that the wait-and-see approach protects 50% of patients from the risk of RT toxicity while also leading to a risk of local treatment failure and progression, with a 5-year LC rate of 100% for patients treated with RT vs. 50% for patients not treated with RT (P<0.02) (4). Radiation as a carcinogenic factor has been clearly demonstrated (37, 38). The risk of radiation-induced tumors in central nervous system has been documented to be about 1-3% (37, 39). Basic criteria for radiation-induced tumor were established by Cahan et al. (40). A meta-analysis showed that the mean latency from RT to secondary tumors was 9 years (41). And radiation-induced tumors after SRS are rare. In our study, 1 patient was treated twice with SRS and the second pathological result was WHO grade IV tumor. It does not exclude radiation-induced secondary tumor after SRS.

In 1997, Schild et al. reported the first case of a patient treated with Gamma Knife SRS after STR, but no efficacy was reported (4). Compared to EBRT, SRS precisely concentrates single high doses of radiation to target and have the rapid radiation fall-off outside the target, leaving surrounding tissue exposed to low dose. SRS is usually finished in fewer fractions while EBRT treatment usually takes several weeks. SRS is suitable because CNs are inert and have clear boundaries with brain parenchyma (9, 42). A multicenter retrospective study showed that LC rates for SRS group, EBRT group were 93% and 88%, respectively (P=0.40) (9). Many studies have shown little neurotoxicity or complications after SRS treatment (43, 44). However, the smaller target area and the lower dose of SRS results in a higher risk of recurrence (9). In our study, 1 patient was treated by Gamma Knife SRS for the second time as salvage therapy. The survival period of 40 months suggests that SRS may be used as an adjuvant treatment after incomplete resection and as salvage therapy.

To date, there have been few reports on the optimal RT dose and delivery device for CNs (45). Rades et al. concluded that for patients treated with STR+RT, a radiation dose of 54 Gy appeared to be sufficient to improve LC, but not statistically significantly (15). In another report, in patients with typical CN, there was no significant difference in OS or LC between those who received a radiation dose of >54 Gy and <54 Gy, whereas for patients with atypical CN who underwent partial resection, the LC rate was better with a radiation dose >54 Gy (P=0.05). Therefore, patients with atypical CN undergoing STR were recommended to receive a radiation dose of 56–60 Gy (22). On the other hand, an international multicentric study showed no significant difference in OS and PFS for radiotherapy dose of 54Gy and greater than 54Gy (P>0.05) (21). In our study, the median EBRT dose was 54.54Gy (46-61.60Gy). Patients who received EBRT had no recurrence. Therefore, we suggest that a dose of 54 Gy may be appropriate for patients with CN.

**Figure 4:**
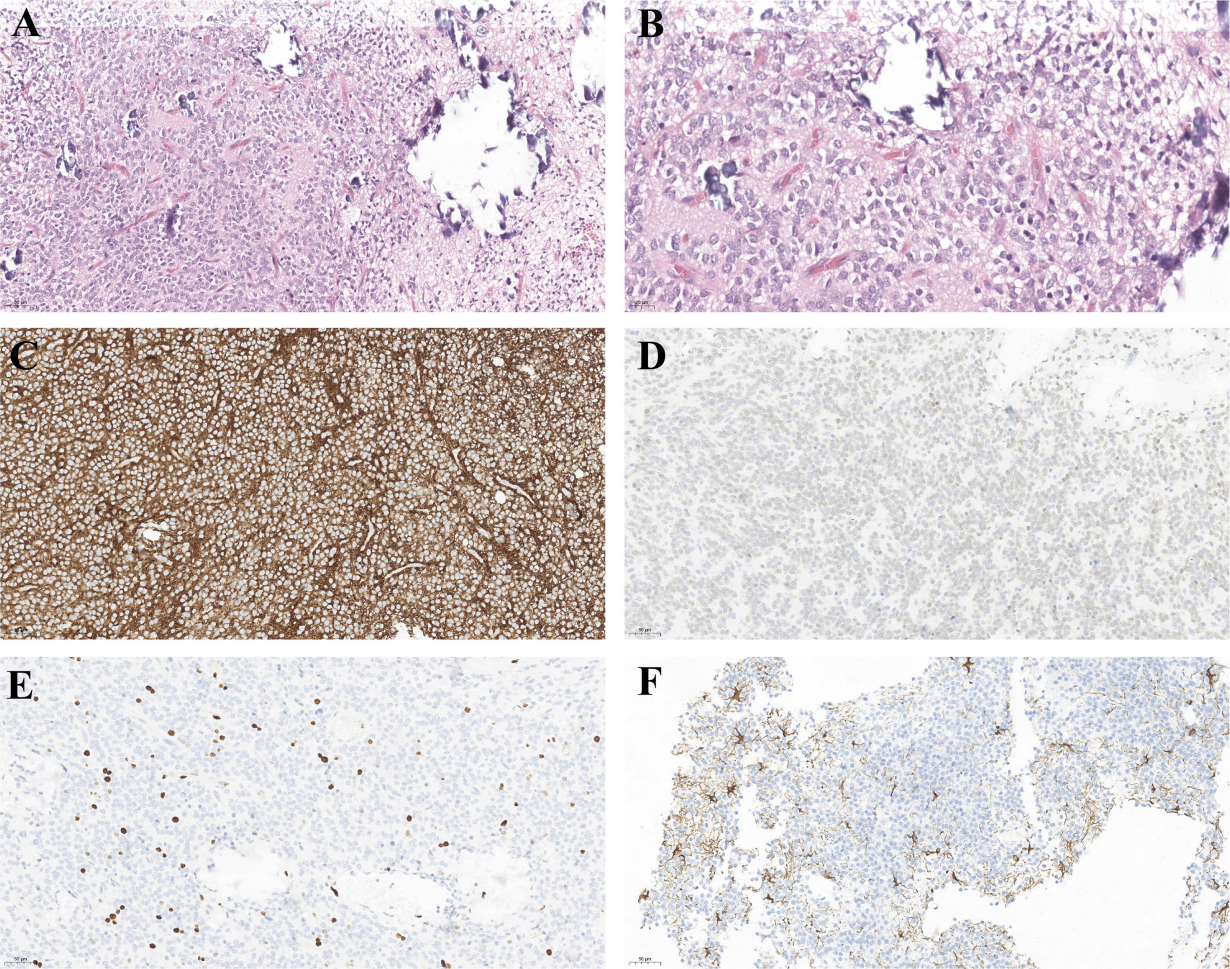
Light microscopy of the surgical specimens reveals small round cells, perinuclear halos in fibrillary neuropils. **(A)** hematoxylin–eosin stain, original magnification ×200. **(B)** hematoxylin–eosin stain, original magnification ×400. And the immunohistochemical features of CN. Tumor cells are positive for SYN **(C)**, NEUN **(D)**. **(E)** Ki-67, **(F)** GFAP is positive in some CNs.

The morphological appearance of CNs tumor cells under light microscopy is uniformly small, round cells of uniform size and shape, with visible calcification and a perinuclear halo around the nucleus due to the lack of cytoplasm. Immunohistochemistry is positive for synaptophysin (Syn), Neuronal Nuclei (NeuN), neuron-specific enolase (NSE), but generally does not express vimentin (Vim), oligodendrocyte transcription factor-2 (Olig-2), and a few Glial fibrillary acidic protein (GFAP). The pathological features of CN are shown in **Figure 4**.

Although CNs are benign tumors, atypical CNs are more aggressive than typical CNs and tend to show a high propensity for recurrence due to their relatively malignant biological behavior. In our long-term follow-up, 1 patient relapsed and developed into a WHO grade IV malignant embryonic tumor, demonstrating its potential malignant biology. Atypical CNs have varying degrees of histological mitosis, vascular proliferation and local necrosis (17). Bertalanffy et al. reported a recurrence rate of 21% (8-43%) for CNs (46). Some studies have suggested that patients with atypical CNs have a worse prognosis and a higher recurrence rate (2, 47). Rades et al. consider proliferative potential as a prognostic factor (15). The most common thresholds for the clinical use of MIB-1 LI in classifying atypical and typical CNs remain controversial, including 2% (48), 3% (49), 4% (50). However, the survival outcomes of these studies (48–50) did not differ significantly between typical and atypical CNs. Consistent with these studies, our study showed that there was no statistically significant difference in OS (P=0.338) and PFS (P=0.277) between typical and atypical CNs.

Whether atypical CNs should receive more treatment remains a topic of debate. A meta-analysis of 310 patients with CN by Rades et al. showed that in patients with atypical CN who underwent STR with RT increased the 5-year LC rate from 5% to 65% and the 5-year OS rate from 46% to 69% (27). Several studies have demonstrated that RT exerts good control over the residual tumor (45, 51). The results of our study are in line with these findings (22, 24). We observed a significant improvement in the OS and PFS of patients with atypical CN who received RT after STR compared to those who received STR alone, and the 5-year PFS improved from 25% to 75% (P=0.004). Therefore, we believe that patients with atypical CN who can only undergo STR will benefit from postoperative RT.

Our multivariate Cox models and competing risk model showed that STR is an independent prognostic factor for poor prognosis in patients with CNs. Consistent with the data published previously, the extent of surgical resection is the most important prognostic factor (15). In our study, the extent of surgical resection was a confounding factor when the effects of RT were examined, and Model 3 showed that postoperative RT significantly improved OS (HR=0.145, 95% CI 0.028–0.746, P=0.021). Furthermore, postoperative RT was also an independent prognostic factor in our competing risk model (P=0.014). Many studies concluded that STR combined with postoperative RT is superior to STR alone, and that it shows no significant difference in OS compared to GTR (21, 22). By combining the two models, we concluded that postoperative RT was an independent prognostic factor affecting for survival.

This study is subject to some limitations. It is a retrospective study with inherent bias; consequently, there may be undetected confounding factors in the subgroup and prognostic analyses, which may affect the statistical models and the results of our analyses. However, there are also some clear advantages to this study. For instance, it is, to the best of our knowledge, the largest retrospective single-center study to date on the long-term survival of patients with CN in China. Also, sensitivity analysis was performed to verify the robustness of the results and evaluate the effects of RT. Therefore, our data will provide a valuable contribution to the existing literature and will support future meta-analyses of this rare tumor.

In summary, the first-choice treatment for CNs is complete surgical resection. The survival rate of incomplete resection followed by adjuvant RT is comparable to that of complete resection. The prognostic value of atypical compared with typical CNs require further investigation, and RT may have a survival benefit on prognosis.

## Conclusion

CNs are a rare type of tumor. This study has analyzed the efficacy of treatment modalities and prognostic factors for CNs. Total surgical resection is recommended whenever possible, provided that side effects can be minimized. Adjuvant RT improves the prognosis of patients who have undergone subtotal resection, especially for atypical CNs, which have a high tendency for recurrence. Modern RT techniques have proven to be effective, and have acceptable levels of associated toxicity. Therefore, it is essential to weigh the pros and cons of available treatment modalities to develop optimal, individualized treatment plans for patients with CN. The results of this study will help to guide the treatment of CNs.

## Data Availability Statement

The raw data supporting the conclusions of this article will be made available by the authors, without undue reservation.

## Author Contributions

QX and RW are responsible for the conception and design; RW is responsible for the administrative support; BX, LO, MW, and ZT are responsible for the provision of study materials or patients; YH, XY, JH, and ZL are responsible for the collection and assembly of data; QXis responsible for the data analysis and interpretation; all authors are responsible for the manuscript writing; and all authors are responsible for the final approval of the manuscript. All authors contributed to the article and approved the submitted version

## Funding

This study was supported by the grants from the Hunan Provincial Natural Science Foundation of China (NO. 2021JJ31104, NO. 2019JJ40497).

## Conflict of Interest

The authors declare that the research was conducted in the absence of any commercial or financial relationships that could be construed as a potential conflict of interest.

## Publisher’s Note

All claims expressed in this article are solely those of the authors and do not necessarily represent those of their affiliated organizations, or those of the publisher, the editors and the reviewers. Any product that may be evaluated in this article, or claim that may be made by its manufacturer, is not guaranteed or endorsed by the publisher.
